# Ground-motion heterogeneity across four subdomains in Yunnan, China revealed by generalized spectral inversion

**DOI:** 10.1038/s41598-025-24103-7

**Published:** 2025-11-18

**Authors:** Ziyan Wang, Xiaojun Li, Lei Fu, Su Chen, Kelin Chen, Yiming He

**Affiliations:** 1 Key laboratory of Urban Security and Disaster Engineering of the Ministry of Education, Beijing University of Technology, Beijing, 100124 China; 2 Institute of Geophysics, China Earthquake Administration, Beijing, 100081 China

**Keywords:** Generalized inversion technique, Empirical reference site, Stress drop, Quality factor, Site amplification, Stochastic finite-fault simulation, Environmental sciences, Natural hazards, Solid Earth sciences

## Abstract

This study investigates regional ground-motion characteristics in Yunnan Province using 1,065 recordings from 60 small-to-moderate earthquakes (3.0 ≤ *M*_w_ ≤ 6.0) at 65 stations. A generalized inversion technique (GIT) using empirical reference site was applied to four tectonically distinct subregions (A–D). The inversion results reveal stress drops (Δ*σ*) ranging from 0.3 to 7.34 MPa, with the highest averages in Regions C and D (2.21 MPa), followed by Region A (2.07 MPa) and Region B (1.16 MPa). Δ*σ* shows no clear dependence on moment magnitude (*M*_w_) or focal depth but correlates with surface heat flow. Reverse-faulting events exhibit a higher average Δ*σ* than strike-slip and normal-faulting events. Quality factor models for the four study areas (A–D) are 118.41*f* ^0.691^, 120.26*f* ^0.638^, 77.20*f* ^0.598^ and 72.19*f* ^0.861^, respectively. The eastern regions (C and D) exhibit lower *Q*_0_ and stronger S-wave attenuation, along with higher tectonic activity and lower surface heat flow. The predominant frequencies of average site amplification for GMX-A, GMX-B, GMX-C and GMX-D are 20.00 Hz, 10.83 Hz, 3.97 Hz, and 1.92 Hz, with corresponding amplification factors of 2.84, 4.31, 5.45, and 7.69. Finally, the inversion parameters were validated through stochastic finite-fault simulations of the 2009 *M*_S_ 6.3 Yao’an mainshock.

**Supplementary Information:**

The online version contains supplementary material available at 10.1038/s41598-025-24103-7.

## Introduction

The Sichuan–Yunnan Block (SYB, 21°N–35°N, 97°E–106°E), situated along the southeastern margin of the Tibetan Plateau, plays a key role in crustal material extrusion driven by the ongoing Indian–Eurasian collision^1–3^. Yunnan Province, the primary focus of this study within the southern SYB, exhibits a pronounced stepwise topographic gradient from northwest to southeast, with elevations ranging from approximately 3,000–4,500 m in the northwest, 2,000–2,500 m in the central, and below 1,500 m in the southern^4,5^. This complex topography results directly from intense crustal compression and uplift associated with active tectonic processes. These processes have produced an extensive fault system, where strain energy accumulates as a result of ongoing plateau uplift and tectonic loading, rendering the region a significant seismic hotspot^6,7^.​

Yunnan Province is transected by several major regional fault zones, including the Nujiang, Lancangjiang, Red River, and Xiaojiang fault zones, which control the primary seismic activity in the surrounding region^8^. As a result of this intense tectonic regime, the region has historically experienced frequent moderate-to-strong earthquakes, such as the 2003 *M*_w_ 6.0 and *M*_w_ 5.6 Dayao earthquakes^9^, the 2014 *M*_w_ 6.2 Ludian earthquake^10,11^, the 2014 *M*_w_ 6.1 Jinggu earthquake^12^ and the 2021 *M*_w_ 6.1 Yangbi earthquake^13,14^. These damaging events have led to substantial social and economic losses. Consequently, conducting comprehensive seismic hazard assessments and constraining region-specific ground-motion parameters is essential for mitigating seismic risk in the region.

The generalized inversion technique (GIT) was first proposed by Andrews (1986)^15^, with the core idea of decomposing observed ground motions in the frequency domain into source, path, and site effects. Subsequently, the parameterized inversion approach was developed: the source term is commonly represented by the classical ω² model^16,17^; the path term was refined by Iwata and Irikura (1986, 1988)^18,19^ through the introduction of geometrical spreading and frequency-dependent anelastic attenuation; and the site term is typically constrained and decoupled from the source by means of reference-site normalization^19^, reference-event normalization^20^, or absolute calibration based on geological and site information^21^. To address the uncertainties associated with fixed model assumptions, Castro et al. (1990)^22^ proposed a non-parametric inversion scheme, in which path attenuation is represented as a discrete function of frequency and distance, This approach, initially implemented in two steps, has since been extended to a one-step formulation that simultaneously resolves source, path, and site effects^23^. More recent studies have examined its robustness under limited data conditions^24^ and explored connections with non-ergodic ground-motion models^25^. Overall, GIT has been widely applied in seismically active regions such as California, Japan, Europe, and China^26–30^.

**Fig. 1:**
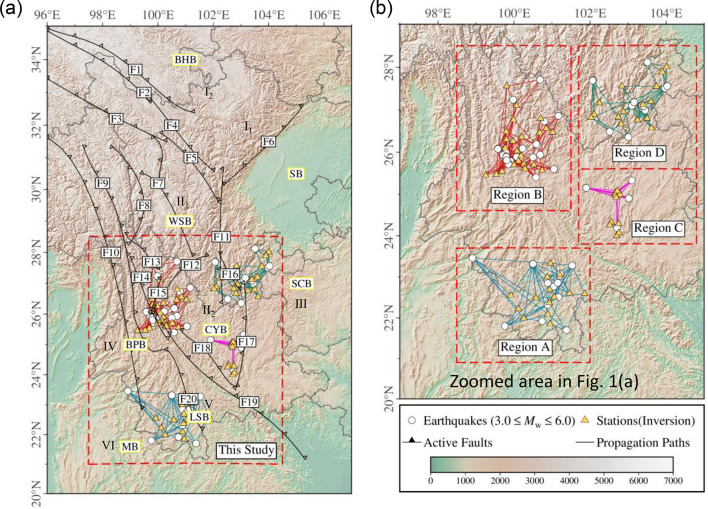
(**a**) The spatial distribution of selected earthquakes (3.0 ≤ *M*_w_ ≤ 6.0), fault zones, and major active tectonic blocks from 2000 to 2020 within the Sichuan–Yunnan Block (SYB), China. Faults are labeled as follows: F1(Dari Fault), F2(Bayan Har Mountains Fault), F3(Ganzi–Yushu Fault), F4(Yuke Fault), F5(Xianshuihe Fault), F6(Longmenshan Fault), F7(Litang Fault), F8(Jinshajiang Fault), F9(Lancangjiang Fault), F10(Nujiang Fault), F11(Anninghe Fault), F12(Xiaojinhe–Lijiang Fault), F13(Deqin–Zhongdian Fault), F14(Weixi–Qiaohou Fault), F15(Longpan–Qiaohou Fault), F16(Zemuhe Fault), F17(Xiaojiang Fault), F18(Chuxiong–Jianshui Fault), F19(Red River Fault), F20(Wuliangshan Fault). (**b**) Delineation of the four subdomains (A–D) and the spatial distribution of strong-motion stations and selected earthquakes within each subdomain. Map generated using Generic Mapping Tools (GMT, version 6.0; https://gmt-china.org/).

The SYB is structurally divided into six first-order tectonic blocks^31,32^: (Ⅰ) the Bayan Har Block (BHB) in the north, which is further subdivided by the Longriba Fault into the Aba Sub-block (Ⅰ_1_) and Maerkang Sub-block (Ⅰ_2_), (Ⅱ) the Sichuan–Yunnan Rhombic Block (SYRB) at the center, which is split by the Xiaojinhe–Lijiang Fault into the Western Sichuan (Ⅱ_1_) and Central Yunnan (Ⅱ_2_) Sub-blocks; and (Ⅲ) the South China Block (SCB) to the east. The southwestern SYB remains contentious, but three primary tectonic units are generally identified: the Baoshan–Puer Block (BPB, Ⅳ), bounded by the Nujiang–Lancangjiang Fault, the Lanping–Simao Block (LSB, Ⅴ), located between the Lancangjiang and Red River Faults, and the Myitkyina Block (MB, Ⅵ), delineated by the Tengchong–Lancangjiang Fault, as shown in Fig. 1(a).

Existing inversion studies in the SYB are primarily concentrated in the Longmenshan fault zone and its surrounding areas^33–39^. In contrast, comprehensive investigations across Yunnan Province remain limited. Earlier studies^40,41^ primarily relied on sparse seismic networks and limited datasets (all from before 2008), resulting in limited regional coverage. More recent studies^42,43^, while utilizing modern strong-motion datasets from upgraded seismic networks, are geographically constrained to localized tectonic domains, such as the Lanping–Simao (Fig. 1(a), LSB) and Baoshan–Puer Blocks (Fig. 1(a), BPB). The absence of province-scale zonation schemes in these studies hinders comparative analysis of inversion parameters across different tectonic subdomains. Additionally, variations in bandwidth filtering ranges and reference station selection criteria introduce systematic biases in the inversion results, and the insufficient number of strong-motion records in Yunnan Province hampers the development of reliable ground motion prediction equations (GMPEs). Collectively, these limitations present substantial challenges to seismic hazard assessments across Yunnan Province.

In this study, Yunnan Province was subsequently divided into four subdomains (A–D; Fig. 1b) based on the spatial distribution of selected strong-motion records, seismic stations, fault zones^31,32^, and the CVM-2.0 velocity model^44^ (Supplementary Fig. S1). The number of stations and events in each subdomain (Regions A–D) of Yunnan Province is summarized in Supplementary Table S1. We then systematically characterize the source, path, and site effects of ground motions within each subdomain. Rather than relying on traditional reference stations, empirical reference site amplification functions were developed for each station, and incorporated into the GIT framework. By decomposing the Fourier Amplitude Spectra (FAS) for these subdomains, the following parameters were derived: (1) source stress drop (Δ*σ*) for each event, (2) frequency-dependent quality factor (*Q*) for each subdomain, (3) station-specific site amplification function, and (4) NEHRP site-class averaged amplification function of Yunnan Province. The inversion results and inter-subdomain differences are discussed, using the inverted parameters to simulate the 2009 Yao’an *M*_S_ 6.3 mainshock, thereby validating the reliability of the inverted parameters and the simulation accuracy.

## Data preprocessing and subdomain delineation

The China Strong Motion Network Center (CSMNC) acquired over 20,000 strong-motion recordings in the SYB from 2008 to 2020, approximately 5,000 of which were collected in and around Yunnan Province. For this study, 1,065 accelerograms from 60 earthquakes (3.0 ≤ *M*_w_ ≤ 6.0) recorded at 65 stations (0 km < *R*_epi_ < 300 km) within Yunnan Province were selected. Each station was required to have recorded at least three events, and each event to have been captured by a minimum of three stations. To reduce the potential impact of soil nonlinearity on site response, recordings with peak ground acceleration (PGA) exceeding 1 m/s² were excluded. Additionally, unreliable data were removed by visual inspection (e.g., delayed S-wave triggers, spikes, high noise levels, and missing components). The original three-component recordings were baseline-corrected and processed using a high-pass filter with a corner frequency of 0.1 Hz.

For each record, the S-wave arrival time was manually identified, and the S-wave end time was determined as the point at which 95% of the cumulative squared acceleration was reached. Only recordings with a duration greater than 3 s and a signal-to-noise ratio (SNR) exceeding 3 were retained^29^. To mitigate high-frequency distortions introduced by rectangular windowing, a 2.5%-tapered Hanning window was applied to the selected S-wave segment^43^. The signals were then transformed into the frequency domain using a Fourier transform, and the FAS were smoothed using the Konno–Ohmachi smoothing function with the bandwidth coefficient b set to 40^45^. The root mean square (rms) of the S-wave FAS from the two horizontal components was subsequently calculated according to Eq. (2.1).

2.1 $$FA{S_{rms}}=\sqrt {\left( {FAS_{{{\text{NS}}}}^{2}\left( f \right)+FAS_{{{\text{EW}}}}^{2}\left( f \right)} \right)/2}$$

in which *FAS*_*rms*_ represents the rms of the Fourier amplitude spectra, *FAS*_NS_ and *FAS*_EW_ are the north-south and east-west components of the FAS, respectively.

**Fig. 2:**
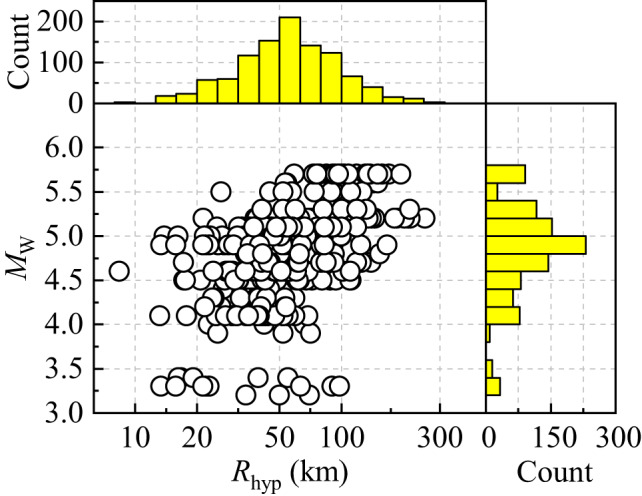
The distribution of S-wave moment magnitudes (*M*_w_) versus hypocentral distances (*R*_hyp_) for 1,065 accelerograms (A: 225; B: 537; C: 102; D: 201) recorded from 60 selected earthquakes across subdomains A–D.

Figure 2 illustrates the distribution of moment magnitude (*M*_w_) versus hypocentral distance (*R*_hyp_) for 1,065 recordings from 60 selected earthquakes. The hypocentral distances (*R*_hyp_) range from 8.39 to 253.81 km, with an average of 63.41 km. Among them, 1,017 records exhibit *M*_w_ values ranging from 3.9 to 5.7. Table 1 summarizes the basic characteristics of the 65 stations across the four subregions. Each station is categorized according to the NEHRP Geomatrix site classification^46^. The numbers of sites in classes A, B, C, and D (hereafter referred to as GMX-A, GMX-B, GMX-C, and GMX-D, respectively) is 2, 9, 28, and 26. The high-frequency attenuation parameter (*κ*) was estimated using the spectral decay method^47,48^. For each record, *κ* was calculated from the average of the two horizontal components. In the *κ*(*R*) regression, epicentral distance was used as the distance metric. The high-frequency decay was fitted within a frequency window [*f*_X_, *f*_Y_], where *f*_X_ was chosen to be larger than the event corner frequency (*f*_0_) to minimize source effects. The ending frequency *f*_Y_ was defined either when the signal-to-noise ratio (SNR) dropped below 3 or when the clear linear fall-off of the spectrum ceased. Following the recommendation of Ktenidou et al. (2014)^49^, the minimum usable bandwidth was set to 10 Hz, and only records with durations longer than 3 s were retained. The *V*_S30​_ values for the stations were obtained from Xie et al. (2022)^50^, and our regression analysis yielded the following site-specific *κ*_0_–*V*_S30_ relationship: *κ*_0_ = 0.0474 + (0.5 × 10^−5^) *V*_S30​_.

**Table 1:** General information of the 65 selected strong motion stations.

Region	Station	Latitude (°)	Longitude (°)	Site class	κ_0_	V_S30_(m/s)	*R*_hyp_ range (km)
A	53JDD	22.37	100.93	C	0.0298	347.68	[29.8, 139.6]
A	53JJH	22.59	101.86	C	0.0235	479.58	[72.0, 111.2]
A	53JMH	21.85	100.95	C	0.0329	292.07	[24.00, 170.1]
A	53JMY	22.09	100.89	D	0.0304	327.82	[26.2, 253.8]
A	53JPW	22.51	101.06	D	0.0338	256.93	[29.7, 107.8]
A	53JZX	23.33	100.96	C	0.0296	352.97	[39.3, 210.4]
A	53LCX	22.55	99.92	C	0.0248	453.33	[104.1, 145.3]
A	53MMM	22.22	100.12	C	0.0314	341.16	[66.6, 185.7]
A	53MMZ	21.99	100.26	D	0.0361	229.35	[50.1, 215.1]
A	53PDH	23.00	100.88	C	0.0315	354.80	[22.7, 73.7]
A	53PMX	23.05	101.21	C	0.0283	387.68	[26.8, 79.7]
A	53SML	22.60	101.46	C	0.0279	384.03	[31.9, 76.5]
A	53SMM	22.69	100.94	B	0.0275	387.90	[31.8, 84.3]
A	53SSM	22.49	100.58	D	0.0317	301.87	[38.7, 202.6]
B	53BBJ	25.70	100.52	D	0.0398	194.36	[23.7, 76.3]
B	53BWY	25.45	99.26	B	0.0304	265.72	[67.4, 73.9]
B	53DFY	25.60	100.30	D	0.0399	191.11	[37.3, 91.2]
B	53DHD	25.72	100.26	D	0.0403	180.66	[32.4, 81.0]
B	53DSL	25.91	100.19	B	0.0294	320.67	[16.7, 66.0]
B	53DWQ	25.79	100.12	D	0.0366	239.73	[16.2, 96.1]
B	53DZF	25.61	100.27	C	0.0318	468.97	[39.8, 88.1]
B	53ENJ	26.26	99.98	C	0.0354	231.75	[36.6, 62.7]
B	53EQH	26.10	99.77	A	0.0273	392.33	[21.4, 140.0]
B	53EYS	26.03	100.08	D	0.0386	175.69	[17.6, 52.3]
B	53HSG	26.36	100.20	C	0.0325	287.83	[30.5, 80.5]
B	53JCS	26.30	99.80	B	0.0268	438.44	[34.4, 48.8]
B	53JGZ	26.80	100.70	C	0.0377	177.33	[57.5, 72.3]
B	53JYC	26.49	99.80	C	0.0280	423.98	[51.2, 90.3]
B	53LJH	26.77	99.99	D	0.0419	168.39	[54.0, 126.0]
B	53LLP	27.10	100.07	D	0.0339	260.67	[21.8, 134.7]
B	53XHD	25.57	100.74	D	0.0395	193.26	[27.0, 153.0]
B	53XXZ	27.56	99.81	D	0.0351	228.11	[40.8, 181.3]
B	53YBD	25.54	99.69	B	0.0290	345.07	[27.3, 61.2]
B	53YBX	25.70	100.00	D	0.0212	655.47	[17.3, 39.7]
B	53YCH	26.46	100.68	D	0.0379	170.22	[34.6, 73.7]
B	53YLD	26.56	100.85	C	0.0313	267.39	[24.2, 71.0]
B	53YPX	25.47	99.54	D	0.0403	185.32	[47.7, 71.0]
B	53YRH	26.47	101.02	D	0.0335	369.97	[38.8, 68.1]
C	53BWL	25.02	102.67	D	0.0467	164.89	[38.9, 79.9]
C	53CBG	24.99	102.79	D	0.0365	196.37	[27.5, 91.9]
C	53CMX	25.08	102.72	D	0.0331	270.94	[48.2, 99.7]
C	53ELK	25.04	102.67	D	0.0379	188.51	[40.0, 79.2]
C	53HTJ	24.98	102.66	D	0.0445	241.22	[39.1, 79.2]
C	53JCX	24.29	102.75	D	0.0333	268.17	[13.4, 13.8]
C	53LZY	24.92	102.69	D	0.0420	139.62	[34.6, 84.5]
C	53TGD	24.00	102.70	C	0.0358	386.15	[22.0, 22.3]
C	53TLS	24.07	102.76	C	0.0361	368.68	[15.7, 16.1]
C	53YYH	24.26	102.52	D	0.0310	274.06	[21.4, 21.7]
D	51BTT	27.56	102.84	C	0.0349	270.64	[52.7, 117.7]
D	51DCY	27.16	102.25	C	0.0268	406.78	[63.5, 90.7]
D	51HDQ	26.67	102.82	C	0.0306	405.01	[38.3, 66.5]
D	51HDX	26.83	102.74	C	0.0325	346.10	[45.3, 118.8]
D	51MNF	28.43	102.17	B	0.0256	431.65	[56.8, 83.9]
D	51MYL	26.89	102.11	C	0.0335	264.27	[61.5, 92.2]
D	51MYS	26.82	102.03	C	0.0330	273.81	[62.2, 214.5]
D	51NNH	26.96	102.88	C	0.0341	283.13	[28.4, 132.3]
D	51YBH	26.53	101.93	B	0.0293	392.98	[59.9, 131.9]
D	53DJL	28.01	104.01	B	0.0237	484.73	[50.8, 57.5]
D	53DSS	27.89	103.90	A	0.0133	696.53	[41.3, 46.9]
D	53HYC	26.80	103.50	B	0.0197	515.79	[23.9, 100.3]
D	53HZH	26.57	103.62	D	0.0351	308.85	[51.1, 82.6]
D	53LDS	27.20	103.60	C	0.0344	243.40	[51.7, 59.9]
D	53LLT	27.12	103.39	C	0.0363	300.22	[8.4, 81.6]
D	53QJX	26.76	103.25	C	0.0328	320.64	[34.2, 118.9]
D	53ZJA	27.55	103.75	C	0.0314	365.37	[26.1, 82.5]

## Method

For a surface ground-motion recording of earthquake *i* at site *j*, the combined *FAS*_*ij*_ of the two horizontal components at a given frequency and hypocentral distance is expressed in Eq. (3.1). The GIT method decomposes the source term *E*_*i*_(*f*), path term *P*(*R*_*ij*_, *f*), and site term *S*_*j*_(*f*) of S-waves in the frequency domain. The problem can be transformed into a linear system by taking the logarithm of both sides of Eq. (3.1), as shown in Eq. (3.6).

3.1 $$FA{S_{ij}}\left( {{R_{ij}},f} \right)={E_i}\left( f \right)P\left( {{R_{ij}},f} \right){S_j}\left( f \right)$$

3.2 $$E\left( f \right)=\left( {{R_{\theta \varphi }}FV/4\pi \rho {\beta ^3}{R_0}} \right) \cdot \left[ {{M_0}{{\left( {2\pi f} \right)}^2}/\left( {1+{{\left( {f/{f_0}} \right)}^2}} \right)} \right]$$

3.3 $$P\left( {R,f} \right)=G\left( R \right){A_n}\left( f \right)=G\left( R \right)\exp \left( { - \pi fR/Q\left( f \right)\beta } \right)$$

3.4 $$G\left( R \right)=\left\{ \begin{gathered} {R^{ - {b_1}}}\:\:\:\:\:\:\:\:\:\:\:\:\:\:\:\:\:\:\:\:\:\:\:\:{\text{ }}R \leqslant {R_1} \hfill \\ R_{1}^{{{b_2} - {b_1}}}{R^{ - {b_2}}}\:\:\:\:\:\:\:\:\:\:\:\:\:\:\:\:\:\:\:\:\:{\text{ }}{R_1} \leqslant R \leqslant {R_2} \hfill \\ R_{1}^{{{b_2} - {b_1}}}R_{2}^{{{b_3} - {b_2}}}{R^{ - {b_3}}}{\text{ }}R \geqslant {R_2} \hfill \\ \end{gathered} \right.$$

3.5 $$S\left( f \right)={S_{ers}}\left( f \right){S_{res}}\left( f \right)$$

3.6 $$\log FA{S_{ij}}\left( {{R_{ij}},f} \right)=\log {E_i}\left( f \right)+\log G\left( {{R_{ij}}} \right) - \frac{{\pi f{R_{ij}}}}{{\ln \left( {10} \right)Q\left( f \right)\beta }}+\log {S_j}\left( f \right)$$

The *FAS*_*ij*_ denotes the rms FAS at frequency *f* for the *i*th event, recorded by the *j*th station at a hypocentral distance (*R*_hyp_). The source term *E*_*i*_(*f*) is constrained by the *ω*^2^ source model, as described in Eq. (3.2)^16^. *R*_*θφ*_ = 0.63 is the average S-wave radiation pattern. *F* = 2 is the free-surface amplification factor. *V* = 0.707 is the partition factor representing the proportion of total S-wave energy in the horizontal components. *ρ* = 2800 kg/m^3^ and *β* = 3.5 km/s represent the average density and S-wave velocity (*V*_*S*_) near the source, respectively^51^. *R*_0_ is a reference distance, commonly set to 1 km. *M*_0_ and *f*_0_ are the seismic moment and the source corner frequency, respectively.

The path term *P*(*R*_*ij*_, *f*) typically consists of geometrical spreading *G*(*f*) and inelastic attenuation *A*_*n*_ (*f*), as described in Eq. (3.3). The geometrical spreading function *G*(*R*) follows the trilinear model proposed by Atkinson and Mereu (1992)^52^, which characterizes S-wave amplitude decay over distinct distance intervals (Eq. 3.4). The coefficients *b*_1​_ = 1.0, *b*_2_ ​= 0, *b*_3 ​_= 0.5, *R*_1_​ = 1.5*H*_Moho_ and *R*_2_​ = 2.5*H*_Moho_ are empirically selected, *H*_Moho_ = 45 km represents the average Moho depth beneath Yunnan Province^53^. The inelastic attenuation function *A*_*n*_(*f*) quantifies the frequency-dependent energy loss of S-waves as they propagate through the medium. This attenuation is governed by the region quality factor *Q*(*f*) = *Q*_0_ *f* ^*α*^, where *Q*_0_ denotes the quality factor at 1 Hz and reflects the heterogeneity of the propagation medium, and the exponent *α* quantifies the frequency dependence of *Q*(*f*).

The site term *S*_*j*_(*f*) is typically decomposed into two components: the empirical reference site amplification *S*_*ers*_(*f*) and the residual site effect *S*_*res*_(*f*)^29^, as expressed in Eq. (3.5). The empirical reference site amplification function *S*_*ers*_(*f*) is generally modeled as the product of an amplification function *A*(*f*) and a diminution function *D*(*f*)^54^, as shown in Eq. (3.7). The residual site effects *S*_*res*_(*f*) accounts for additional amplification caused by other mechanisms, such as local topographical effects, basin effects, and energy losses due to reflection, refraction, and scattering. After correcting the site-specific *S*_*ers*_(*f*), the resulting *S*_*res*_(*f*) fluctuates around 1 across the entire frequency band. When a sufficient number of stations are available, it can be reasonably assumed that the sum of the logarithms of all *S*_*res*_(*f*) values is approximately zero^38^, as shown in Eq. (3.8).

3.7 $${S_{ers}}\left( f \right)=A\left( f \right)D\left( f \right)=\sqrt {{\rho _s}{\beta _s}/\overline {Z} \left( f \right)} \exp \left( { - \pi f{\kappa _0}} \right)$$

3.8 $$\sum\limits_{{j=1}}^{N} {\log \left( {{S_{res,j}}\left( f \right)} \right)} =0$$

3.9 $$\overline {Z} \left( f \right)=\int\limits_{0}^{{z\left( f \right)}} {\rho \left( z \right)} dz/\int\limits_{0}^{{z\left( f \right)}} {\frac{1}{{\beta \left( z \right)}}} dz$$

3.10 $$\rho \left( z \right)=\overline {\rho } \left( 0 \right)+\left[ {{V_S}\left( z \right) - \overline {{{V_S}}} \left( 0 \right)} \right]\frac{{{\rho _c} - \overline {\rho } \left( 0 \right)}}{{{\beta _c} - \overline {{{V_S}}} \left( 0 \right)}}$$

The amplification function *A*(*f*) is defined as the square root of the seismic impedance ratio between the source and the surface. *ρ*_*s*_ and *β*_*s*_ represent the density and *V*_*S*_ near the source, respectively. The average near-surface seismic impedance $\overline {z}(f)$ is a function of frequency , as shown in Eq. (3.9). The upper limit of the integral, *z*(*f*) = *β*/4*f*, corresponds to the depth of one-fourth the seismic wavelength^54^. *ρ*(*z*) and *β*(*z*) represent the density and *V*_*S*_ at depth *z* in the constructed model. *ρ*(*z*) is defined by an empirical density model proposed by Boore and Joyner (1997)^55^, as shown in Eq. (3.10). $\overline {\rho}(0)$ = 1700 kg/m^3^ and $\overline {V}_s(0)$ = 1300 m/s represent the average density and *V*_*S*_ at the earth’s surface. *ρ*_*c*_ = 2800 kg/m^3^ and *β*_*c*_ = 3500 m/s represent the average crustal density and *V*_*S*_ in Yunnan province^29^.

## Result

### Empirical reference site

**Fig. 3:**
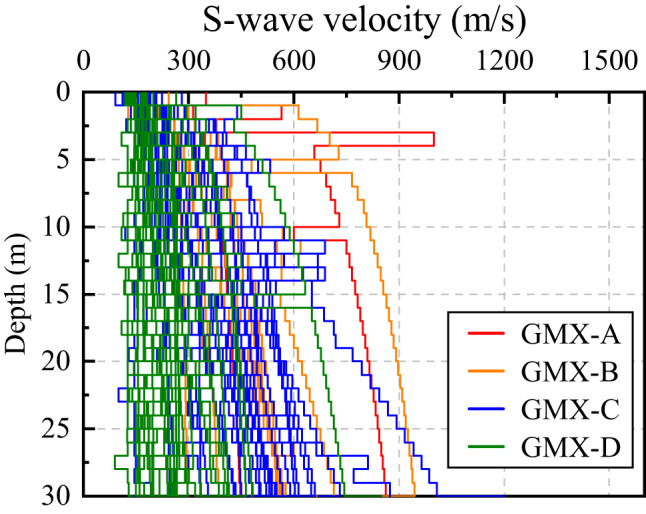
S-wave velocity model (SVM) for the uppermost 30 m at the 65 stations.

**Fig. 4:**
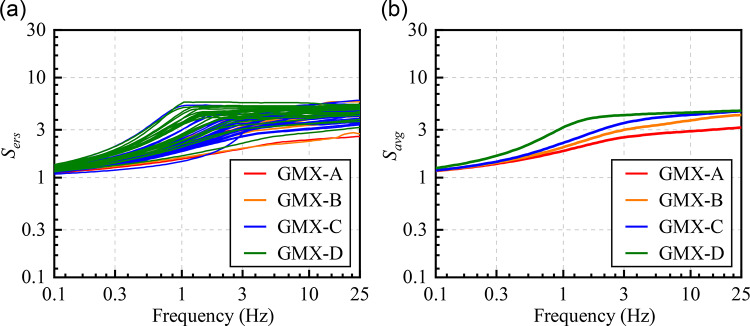
(**a**) Empirical reference site amplification at 65 stations derived using the QWA. (**b**) Average empirical reference site amplification for GMX-A, GMX-B, GMX-C and GMX-D.

Traditional GIT methods for reference sites typically assume a constant site amplification factor of 1 across all frequencies at the bedrock site^38,56^. This assumption requires identifying a bedrock reference site prior to use, which is often challenging under real-world conditions. In fact, even hard bedrock sites typically do not exhibit an amplification factor of 1 due to surface weathering and fracturing^38,57^. To address this limitation, we first constructed *V*_*S*_ models (SVMs) for 65 stations using borehole data (0–30 m depth) from Xie et al. (2022)^50^ and additional borehole data collected in Yunnan Province (Fig. 3). The mid-to-lower crustal and upper mantle *V*_*S*_ profile at depths of 5–25 km were derived from the CVM-2.0 velocity model^44^ by using the *V*_*S*_ value at the nearest grid point to each station. For the intermediate depth range of 0.03–5 km, where observational data were lacking, an exponential function was fitted to each station individually to ensure continuity in the velocity structure. Based on the SVMs, we then calculated the amplification function *A*(*f*) at each station using the quarter-wavelength approximation method (QWA)^55^, which estimates the site transfer function from the impedance contrasts of successive layers. The diminution function *D*(*f*) was determined from the high-frequency attenuation factor (*κ*_0_) following Anderson and Hough (1984)^58^. The formulas for *A*(*f*) and *D*(*f*) are given in Eq. (3.7), and each parameter has been explained in detail in the preceding text. The combined effect of *A*(*f*) and *D*(*f*) approximates the first-order site transfer function, which is commonly used as an approximation of the site response^54^. This approximation remains valid even in the absence of strong-motion recordings, as it allows for the estimation of site effects based on theoretical models or empirical relationships. Figure 4 shows the empirical reference site amplification *S*_*ers*_(*f*) for 65 stations, along with the average amplification *S*_*avg*_(*f*) for site categories GMX-A, GMX-B, GMX-C, and GMX-D. It is evident that softer sites tend to exhibit greater amplification.

### Inversion uncertainties

**Fig. 5:**
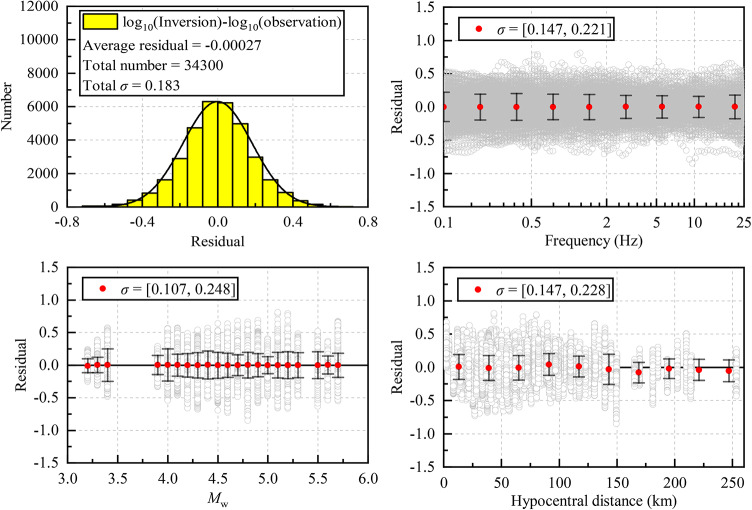
Logarithmic residuals between inverted and observed Fourier amplitude spectra.

The inverted FAS of all strong-motion records were obtained by multiplying the inverted source spectrum, quality factor, and local site amplification. These inverted FAS were then compared with the observed FAS to calculate the logarithmic residuals, as shown in Fig. 5. The histogram of logarithmic residuals exhibits a normal distribution, with a mean of −0.00027 and a standard deviation of 0.183. The logarithmic residual deviations between observed and inverted FAS show no statistically significant dependence on frequency (0.1–25 Hz), moment magnitude (3.0–6.0), or hypocentral distance (0–260 km). The corresponding residual standard deviations range from 0.147 to 0.221, 0.107 to 0.248, and 0.147 to 0.228 for these parameters, respectively. These values are smaller than those reported by Fu et al. (2023)^29^, suggesting that, in our study, using station-specific empirical reference site amplification may provide more accurate initial constraints for the inversion process and thereby reducing systematic errors in the spectral matching.

### Source parametrization

The seismic moment (*M*_0​_) and corner frequency (*f*_0_)​ were estimated using the *ω*² source model^16^, and the Δ*σ* for each event was calculated using Eq. (4.1), where *M*_0_, *f*_0_, and *β* are consistent with Eq. (3.2). The surface-wave magnitude (*M*_S_) and moment magnitude (*M*_w_) for each earthquake were obtained from the National Earthquake Data Center of China (NEDC)^59^ and the U.S. Geological Survey (USGS)^60^. The focal mechanisms of the selected events were compiled from NEDC and Global Centroid Moment Tensor (CMT) project^61^, in which SS represent the strike-slip faulting, NF represent the normal faulting, and RF represent the reverse faulting. The stress parameter estimation results are summarized in Table 2.

4.1 $$\Delta \sigma =\frac{7}{{16}}{M_0}{\left( {\frac{{2\pi {f_0}}}{{2.34\beta }}} \right)^3}$$

**Table 2:** Stress parameter Estimation results of the 60 selected earthquakes.

Region	Origin Beijing Time (yyyy/mm/dd hh: mm: ss)	Lat (°)	Lon (°)	Depth (km)	M_S_	M_w_	Faulting style	M_0_ (*N*·m)	f_0_ (Hz)	Δσ (MPa)
A	2007/06/03 05:42:00	22.85	100.89	15	4.3	4.0	–	1.12 × 10^15^	1.11	0.62
A	2007/06/03 06:27:43	22.77	101.05	26	3.8	4.1	–	1.58 × 10^15^	1.20	1.40
A	2007/06/03 08:09:37	22.86	101.15	15	4.1	4.4	–	4.47 × 10^15^	0.63	0.58
A	2007/06/03 10:49:01	23.03	101.21	11	4.9	4.9	SS	2.51 × 10^16^	0.51	1.89
A	2012/07/30 00:05:33	23.08	101.22	10	4.2	4.3	SS	3.16 × 10^15^	0.66	0.53
A	2014/01/28 20:01:59	22.51	101.17	7	4.7	4.5	SS	6.31 × 10^15^	0.80	1.87
A	2014/12/06 02:43:45	23.32	100.49	10	5.9	5.6	SS	2.82 × 10^17^	0.26	1.61
A	2014/12/06 18:20:00	23.32	100.50	10	5.9	5.6	SS	2.82 × 10^17^	0.26	2.22
A	2015/03/01 18:24:40	23.46	98.91	11	5.5	5.2	RF	7.08 × 10^16^	0.54	6.66
A	2016/03/05 19:20:24	21.70	101.38	10	4.6	4.5	SS	6.31 × 10^15^	0.66	1.33
A	2016/07/29 22:02:52	21.80	99.76	30	4.7	4.7	–	1.26 × 10^16^	0.96	5.64
A	2018/02/09 22:58:05	22.32	100.89	12	4.9	4.6	–	8.91 × 10^15^	0.61	1.72
A	2018/05/20 07:12:36	21.92	100.74	8	4.2	4.2	–	2.24 × 10^15^	1.57	1.82
A	2018/09/08 10:31:29	23.28	101.53	11	5.9	5.7	SS	3.98 × 10^17^	0.23	1.03
B	2008/02/18 10:44:46	25.79	99.91	14	4.6	4.2	–	2.24 × 10^15^	1.02	1.59
B	2009/04/14 04:37:10	25.99	99.79	10	4.6	4.9	NF	2.51 × 10^16^	0.51	0.68
B	2009/07/09 19:19:14	25.60	101.03	10	6.3	5.7	SS	6.98 × 10^17^	0.310	4.11
B	2009/07/10 17:02:01	25.60	101.05	10	5.4	5.2	SS	7.08 × 10^16^	0.40	1.10
B	2009/11/02 05:07:16	25.94	100.69	10	5.0	4.9	SS	2.51 × 10^16^	0.37	1.17
B	2012/06/24 15:59:34	27.71	100.69	11	5.7	5.5	NF	2.00 × 10^17^	0.15	0.49
B	2013/02/22 05:43:41	26.73	100.80	14	4.2	4.3	SS	3.16 × 10^15^	0.86	1.21
B	2013/03/03 13:41:16	25.93	99.72	9	5.5	5.2	NF	7.08 × 10^16^	0.43	1.58
B	2013/04/17 09:45:54	25.90	99.80	11	5.1	5.1	–	5.01 × 10^16^	0.60	1.15
B	2013/11/28 16:23:54	25.40	100.58	10	4.7	4.9	NF	2.51 × 10^16^	0.29	0.30
B	2014/01/15 03:17:47	26.86	101.17	33	4.5	4.8	RF	1.78 × 10^16^	0.49	0.57
B	2014/08/21 12:11:02	25.88	100.50	12	4.2	4.3	SS	3.16 × 10^15^	0.66	0.50
B	2016/02/08 07:39:08	26.09	99.58	17	4.0	3.9	–	7.94 × 10^14^	1.01	0.76
B	2016/05/18 00:48:48	26.08	99.58	17	5.1	4.9	SS	2.51 × 10^16^	0.48	1.30
B	2016/05/18 01:05:12	26.08	99.58	10	4.6	4.8	NF	1.78 × 10^16^	1.02	1.75
B	2016/11/17 12:10:30	25.74	99.86	10	4.4	4.1	SS	1.58 × 10^15^	1.54	1.91
B	2016/11/17 12:22:48	25.71	99.86	10	4.5	4.5	SS	6.31 × 10^15^	0.69	0.77
B	2017/03/27 07:40:29	25.87	99.81	10	4.9	4.6	SS	8.91 × 10^15^	0.52	0.58
B	2017/03/27 07:55:01	25.79	99.80	12	5.1	5.0	SS	3.55 × 10^16^	0.22	0.37
B	2017/03/27 09:10:26	25.81	99.81	10	4.4	4.1	–	1.58 × 10^15^	1.03	1.06
B	2018/02/15 11:27:41	27.24	99.98	12	4.0	4.2	–	2.24 × 10^15^	0.99	0.65
B	2019/01/08 01:15:36	26.09	100.23	5	3.5	4.5	–	6.31 × 10^15^	0.98	0.91
B	2019/07/21 20:23:28	26.16	100.62	10	4.9	4.8	SS	1.78 × 10^16^	0.39	0.96
B	2019/07/21 22:51:55	26.19	100.59	14	3.4	–	–	7.08 × 10^13^	1.91	2.21
B	2019/08/29 15:30:35	25.89	100.07	11	3.4	–	–	1.41 × 10^14^	1.46	1.29
C	2008/12/26 02:19:55	24.90	103.02	11	4.3	4.1	–	1.58 × 10^15^	1.12	1.55
C	2012/10/15 07:07:59	25.15	101.90	10	4.4	4.7	SS	1.26 × 10^16^	1.34	5.02
C	2015/03/09 17:59:42	25.33	103.10	12	4.5	4.6	SS	8.91 × 10^15^	1.00	3.47
C	2018/08/13 01:44:24	24.19	102.71	7	4.7	5.0	SS	3.55 × 10^16^	0.46	1.62
C	2018/08/13 01:48:17	24.19	102.71	6	3.3	–	–	1.00 × 10^14^	1.19	0.69
C	2018/08/14 03:50:36	24.19	102.71	6	5.0	4.9	SS	2.51 × 10^16^	0.47	0.88
D	2012/09/07 11:19:42	27.51	103.97	14	5.7	5.5	SS	2.00 × 10^17^	0.23	1.42
D	2012/09/07 12:16:30	27.56	104.03	14	5.6	5.3	RF	1.00 × 10^17^	0.43	3.18
D	2012/11/14 19:42:56	26.49	102.52	13	4.2	4.7	SS	1.26 × 10^16^	1.29	2.84
D	2013/02/19 10:47:00	27.10	103.10	10	4.9	4.9	SS	2.51 × 10^16^	0.65	2.12
D	2013/11/16 23:36:41	26.36	103.01	10	4.6	4.6	SS	8.91 × 10^15^	0.64	0.87
D	2014/08/08 14:39:29	27.11	103.34	7	3.9	4.6	SS	8.91 × 10^15^	1.45	1.43
D	2014/08/10 12:39:12	27.04	103.38	10	4.0	4.1	RF	1.58 × 10^15^	0.81	0.53
D	2014/08/17 06:08:00	28.12	103.51	7	5.2	5.0	SS	3.55 × 10^16^	0.67	3.03
D	2014/09/10 16:59:30	27.11	103.36	17	4.3	4.7	–	1.26 × 10^16^	0.97	1.13
D	2016/08/12 19:25:21	26.99	103.45	10	4.4	4.6	SS	8.91 × 10^15^	0.48	0.46
D	2017/02/08 19:11:39	27.09	103.37	10	4.9	4.9	SS	2.51 × 10^16^	0.78	3.19
D	2017/03/12 20:21:18	27.09	103.41	10	4.5	4.8	SS	1.78 × 10^16^	1.05	7.34
D	2018/10/31 16:29:55	27.70	102.08	19	5.1	5.1	SS	5.01 × 10^16^	0.75	3.18
D	2018/11/20 06:01:11	27.69	102.07	14	3.9	–	NF	1.00 × 10^14^	1.19	1.45
D	2020/05/18 21:47:59	27.18	103.16	8	5.0	5.1	SS	5.01 × 10^16^	0.31	0.92

Stress drop (Δ*σ*) quantifies the relationship between the average fault slip and rupture area, which is typically modeled as a circular fault with radius *r* based on the *ω*^2^ source model^16^. For a given rupture radius, Δ*σ* increases with slip magnitude. However, observed Δ*σ* values exhibit significant variability, and different studies on the same earthquake often report inconsistent results^62^, also with respect to a possible Δ*σ* variation with depth^63^. Empirical studies indicate that Δ*σ* is generally constrained within the range of 0.1 to 100 MPa worldwide. Early studies by Kanamori and Anderson (1975)^64^ on global earthquakes (*M* ≥ 6.0, 1923–1968) reported Δ*σ* values ranging from 0.1 to 10.0 MPa, with an average of 6 MPa. Subsequent investigations by Purcaru and Berckhemer (1978)^65^ on large earthquakes (*M* ≥ 7.0, 1857–1976) constrained Δ*σ* within 2 to 13 MPa. Zang (1984)^66^ further emphasized that for most shallow-focus earthquakes, Δ*σ* typically ranges from 2 to 6 MPa. A comprehensive study by Allmann and Shearer (2009)^67^ analyzing 2000 global earthquakes (*M* ≥ 5.0, 1900–2007) revealed Δ*σ* values spanning from 0.3 to 50 MPa, with a median of 4 MPa. In continental China, Zhao et al. (2011)^68^ reported Δ*σ* values ranging from 0.1 to 20 MPa for 2573 small-to-moderate earthquakes (3.0 ≤ *M*_L_ ≤ 6.0), with the majority of events below 10 MPa. The primary factors influencing Δ*σ* include seismic moment (*M*_0_), hypocentral depth, focal mechanism, tectonic setting, and fault properties.

**Fig. 6:**
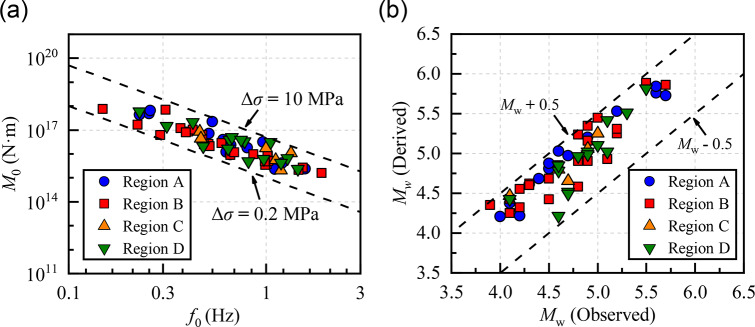
(**a**) Scaling of seismic moment (*M*_0_) versus source corner frequency (*f*_0_). (**b**) Comparison of the *M*_w_ derived from *M*_0_ and observed *M*_w_ obtained from the NEDC and USGS.

Figure 6(a) illustrates a log-linear relationship between *M*_0_ and *f*_0_, described by the scaling relationship log(*M*_0_) = 15.89–2.65log(*f*_0_). For all 60 events, the inverted Δ*σ* values range from 0.3 to 7.34 MPa, with a mean of 1.74 MPa and a standard deviation in logarithmic scale of log_10_(0.16). The inverted mean Δ*σ* values exhibit regional variation (Region C = Region D > Region A > Region B) across the four subdomains: Region A (14 events), 2.07 ± log_10_ (0.37) MPa; Region B (25 events), 1.16 ± log_10_(0.18) MPa; Region C (6 events), 2.21 ± log_10_(0.74) MPa; Region D (15 events), 2.21 ± log_10_(0.39) MPa. Previous studies in Yunnan reported Δ*σ* values primarily in the range of 0.1–10 MPa for small-to-moderate earthquakes, with mean values ranging from 2.3 to 5.05 MPa^69^ and most events below 10 MPa^68^, which encompass the range observed in this study (0.3–7.34 MPa). High Δ*σ* events are primarily concentrated in the Sichuan-Yunnan border zone and the northeastern margin of the Longmenshan Fault, which is consistent with the spatial distribution of our results. Figure 6(b) shows the consistency between the inverted moment magnitudes (*M*_w_) derived from *M*_0_ using Eq. (4.2) and the observed values, with all events exhibiting deviations within ± 0.5 magnitude units​​, confirming the accuracy of our source spectrum fitting parameter *M*_0_.

4.2 $$\log {M_0}=1.5{M_{\text{w}}}+16.05$$

The relationship between Δ*σ* and the faulting mechanism remains a matter of debate. Some studies suggest that reverse faulting events have higher Δ*σ* than strike-slip events, which in turn exhibit higher values than normal faulting events, possibly due to greater frictional strength and shear stress in reverse faults^70–72^. In contrast, other studies have found that strike-slip events exhibit significantly higher Δ*σ* than both reverse and normal faulting events^67,73^. In this study, six earthquakes are classified as normal faulting (NF) with Δ*σ* ranging from 0.30 to 1.75 MPa (average: 1.04 MPa), four as reverse faulting (RF) with Δσ ranging from 0.53 to 6.66 MPa (average: 2.74 MPa), and thirty-three as strike-slip (SS) with Δσ ranging from 0.37 to 7.34 MPa (average: 1.89 MPa). On average, RF events exhibit the highest Δ*σ*, followed by SS and NF events. However, owing to the relatively limited number of NF and RF events in our dataset, the statistical robustness of this result may be limited. Zhao et al. (2011)^68^ analyzed 74 earthquakes (3 ≤ *M*_L_ ≤ 6.0) in mainland China and found no significant correlation between Δ*σ* and focal mechanism, highlighting the complexity of this relationship.

**Fig. 7:**
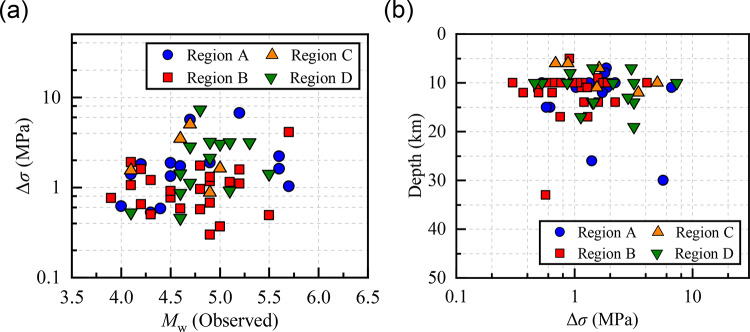
(**a**) The Δ*σ* versus *M*_w_ obtained from the NEDC and USGS. (**b**) The Δ*σ* versus focal depth.

As shown in Fig. 7, no significant correlations are observed between Δ*σ* and either *M*_w_or focal depth in our dataset, consistent with previous studies^73–75^. These results suggest that Δ*σ* is generally independent of *M*_w_, likely due to differences in rupture characteristics between small and large events^76^. Uchide et al. (2014)^77^ reported a positive Δ*σ*–depth correlation at depths of 30–60 km for 3.0 ≤ *M*_w_ ≤ 4.5 earthquakes prior to the 2011 Tohoku-oki earthquake. In contrast, no significant dependence was observed for events shallower than 30 km or deeper than 60 km, consistent with our results.

Oth (2013)^78^ analyzed 3,964 Japanese earthquakes (2.7 ≤ *M*_w_ ≤ 7.9, 1996–2011) and identified a systematic correlation between Δ*σ* and surface heat flow. In Honshu, ​​low Δ*σ* values (∼0.1–3 MPa) are spatially correlated with high heat flow zones (q_s_ > 100 mW/m^2^)​​, while Kyushu shows higher average Δ*σ* (∼3–20 MPa) with ​​lateral variability linked to heat flow gradients​​ (q_s_ < 60 mW/m^2^). According to surface heat flow data in Yunnan province^40,79^, Region B is located within a high heat flow zone (q_s_ > 80 mW/m^2^) in northwest Yunnan, which may explain its lower mean Δ*σ* (1.16 MPa). Region C is located in the southern part of the Central Yunnan sub-block, where the geothermal heat flow is lower (55–65 mW/m^2^), comparable to Regions A and D. This may account for the relatively higher average Δ*σ* values (2.21 MPa) in these three regions compared to region B.

### Quality factor

**Fig. 8:**
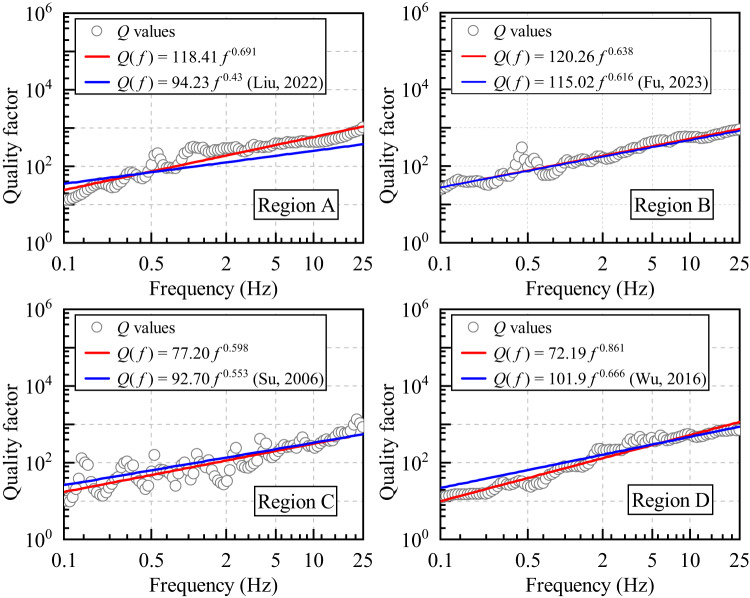
Quality factors (*Q*) and the fitted *Q*(*f*) models for four subdomains.

The quality factor (*Q*) exhibits systematic spatial variations across the study area, with distinct attenuation patterns emerging through comparison with previous studies, as shown in Fig. 8. In region A, the obtained *Q*(*f*) = 118.41*f* ^0.691^ demonstrates elevated *Q*_0_ (118.41) and stronger frequency dependence (*α =* 0.691) compared to *Q*(*f*) = 94.23*f* ^0.43^ from southwestern Yunnan43, derived from 242 three-component strong-motion records (2007–2019). In region B, the obtained *Q*(*f*) = 120.26*f* ^0.638^ aligns closely with *Q*(*f*) = 94.23*f* ^0.43^ along the southwestern margin of SYRB^29^, derived from 42 small-to-moderate earthquakes (2008–2017). Region C demonstrates lower *Q*_0_ (77.20) and similar exponent *α* (0.598) compared to the inversion result (*Q*(*f*) = 92.70*f* ^0.553^) of Su et al. (2006)^40^ in the Central Yunnan sub-block. Region D demonstrates lower *Q*_0_ (72.19) and higher exponent *α* (0.861) compared to the inversion result of Wu et al. (2016)^36^ in Panxi region, which is located adjacent to and to the upper left of region D.

Conventional seismotectonic studies suggest higher *Q* values in tectonically stable regions compared to areas with intense tectonic activity^80^, while regions exhibiting enhanced thermal activity demonstrate further *Q* reduction through viscoelastic dissipation mechanisms^81^. The spatial heterogeneity of *Q* across Yunnan province and its subregions reveals distinct regional characteristics. Our results demonstrate that western Yunnan (Regions A and B), which mainly comprises the Baoshan-Puer and Lanping-Simao blocks, exhibits a relatively low-velocity zone in the upper crust. However, no obvious low-velocity zones are observed in the middle or lower crust. The eastern part (regions C and D) mainly covers the Central Yunnan sub-block, which is flanked on the west by the Jinshajiang-Honghe fault zone, dividing the eastern and western parts of Yunnan province. This may be the main reason for the higher *Q* values in western Yunnan (Regions A and B) than in eastern Yunnan (Regions C and D), which can also be corroborated with the findings of Su et al. (2006)^40^. The average lengths of the propagation paths in the region A–D are 83.74, 55.33, 43.25, and 59.59 km, respectively. Different regional sizes, propagation path lengths, and geothermal heat flow also have some effects on the differences in *Q* values, but in general, such regional differences in *Q* values of Yunnan province are in good agreement with the variability in tectonic activity, seismicity, crustal velocity structure, and geothermal activity states.

### Site amplification

**Fig. 9:**
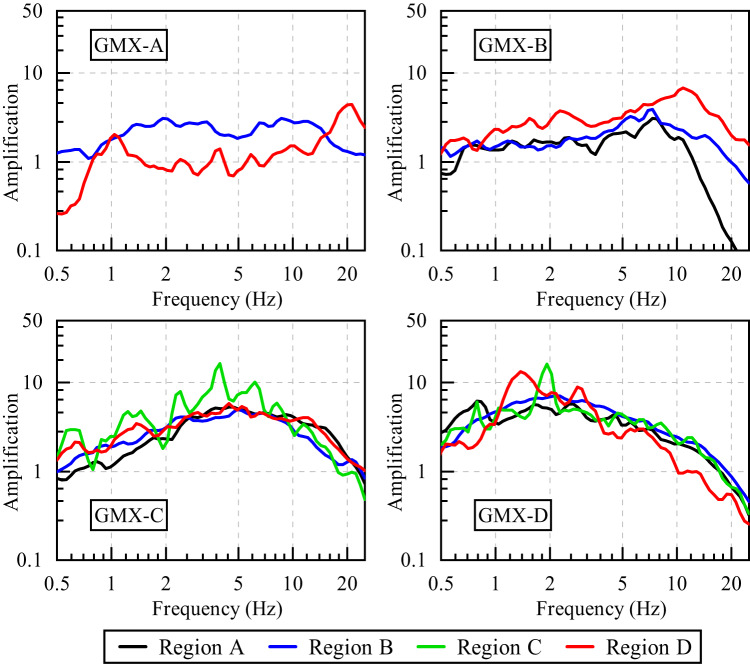
Comparison of the average site amplification of GMX-A, GMX-B, GMX-C, and GMX-D for Regions A–D.

Figure 9 presents the average site amplification effects of GMX-A, GMX-B, GMX-C, and GMX-D for Regions A–D. The numbers of sites in each category are 2, 9, 26, and 23, respectively. GMX-A sites exhibit overall low amplification levels across the frequency range, with amplification factors typically ranging from ~ 1 to 2. Region B (blue) and Region D (red) exhibit relatively flat amplification spectra, indicating the absence of strong site resonance. A slight amplification rise in Region D is observed near 20 Hz, where the amplification factor briefly exceeds a value of 3. This localized high-frequency peak is not typical of rock or very stiff soil and may be attributed to surface-layer effects such as weathered rock or thin sedimentary cover over a stiff base. GMX-B sites exhibit a more pronounced frequency-dependent amplification pattern. Region D (red) displays a distinct peak reaching nearly 8 at frequencies around 10 Hz, indicating significant site resonance. This behavior suggests the presence of a strong impedance contrast between surface and underlying layers, such as a soft-to-stiff soil transition or sedimentary layering that facilitates resonance. Meanwhile, Region A (black) shows a relatively flat response below 5 Hz but a sharp drop-off in amplification beyond this frequency, possibly due to high damping in the upper soil layers or a generally stiffer soil column that suppresses high-frequency wave amplification.

GMX-C sites exhibit significantly elevated amplification across a broad frequency range of approximately 1–10 Hz. Region C (green) in particular demonstrates multiple amplification peaks, with values exceeding 10 in the range of 2–5 Hz. These pronounced and wideband amplification features suggest the presence of thick soft sediment layers or potential basin-edge effects, both of which are known to amplify site response at intermediate frequencies. Compared to Region C, the other regions—A (black), B (blue), and D (red)—exhibit more moderate amplification levels, typically remaining under a factor of 10. Although some fluctuation is still present, especially in the 1–5 Hz range, interregional variations become less pronounced at higher frequencies (> 10 Hz), where amplification curves tend to converge, suggesting similar near-surface conditions. GMX-D sites exhibit the strongest amplification. All regions display a clear amplification peak between 1 and 2 Hz, with Region C again showing the highest values, exceeding a factor of 10. Region D (red) also exhibits substantial amplification, though slightly lower than Region C. This behavior is typical of very soft soil deposits or deep alluvial basins, where strong impedance contrasts between soft surface layers and underlying stiffer strata lead to resonance effects at low frequencies. Region A (black) and Region B (blue) display relatively flatter and lower amplification curves, indicating stiffer ground conditions or thinner soft-soil layers in these areas.

**Fig. 10:**
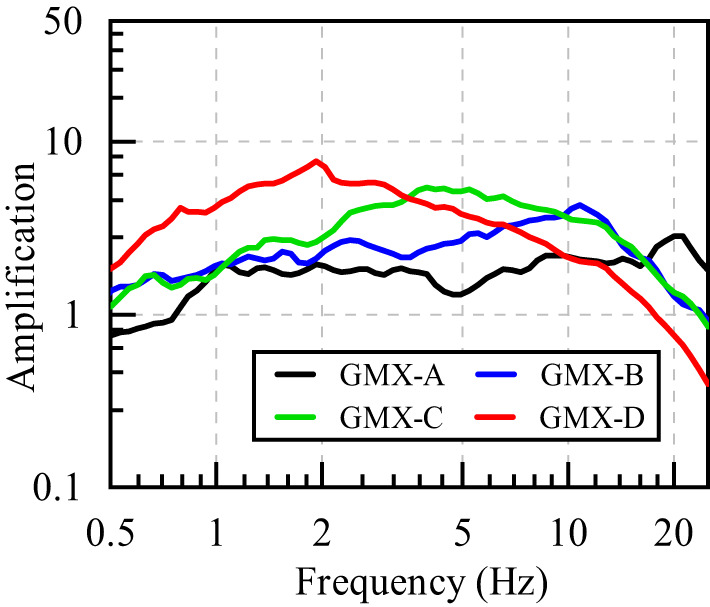
Comparison of the average site amplification of GMX-A, GMX-B, GMX-C and GMX-D for all sites.

Figure 10 presents the average site amplification for GMX-A, GMX-B, GMX-C and GMX-D, categorized according to the criteria outlined in Supplementary Table S2. The corresponding predominant frequencies for these sites are 20.001 Hz, 10.830 Hz, 3.969 Hz, and 1.922 Hz, with amplification factors of 2.843, 4.312, 5.451, and 7.694, respectively. The results indicate that stiffer sites tend to exhibit higher predominant frequencies. A distinct trend reveals that the peak plateau region of the average amplification curves gradually shifts toward lower frequencies as site stiffness decreases. Notably, softer sites exhibit stronger amplification of long-period seismic motions. These observations are consistent with general rules regarding site response characteristics.

### Parameters validation via stochastic ground motion simulation

Fu et al. (2023)^29^ proposed a method that combines GIT and stochastic simulation. This approach, based on the assumption of seismic self-similarity, treats stress drop, quality factor, and site transfer function as representative parameters of a region. As an example, the simulation results were compared with observed data from the Yangbi earthquake, demonstrating good agreement, particularly for periods longer than 0.1 s. This suggests that the GIT inversion parameters are appropriate and reliable for simulating strong ground motion within a region. To further validate inversion parameters of our study, we conducted a stochastic finite-fault simulation using the 2009 *M*_S_ 6.3 Yao’an earthquake as a case study. This event occurred on 9 July 2009 at 19:19 local time (Beijing Time) in Yao’an County, Yunnan province (25.60°N, 101.03°E), corresponding to Region B in this study. The Yao’an earthquake occurred within the seismogenic zone of the 15 January 2000 *M*_S_ 6.5 Yao’an earthquake, with their epicenters located less than 10 km apart.

**Fig. 11:**
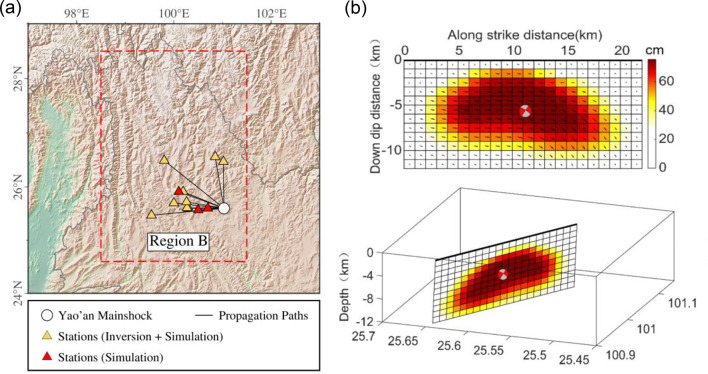
(**a**) Geographical distribution of the 12 selected stations and the 2009 *M*_S_ 6.3 Yao’an mainshock. (**b**) Source slip model of the 2009 *M*_S_ 6.3 Yao’an earthquake inverted using Interferometric Synthetic Aperture Radar (InSAR) observations by Qu et al. (2019)^82^.

Based on the slip distribution models of the Yao’an earthquake obtained from Synthetic Aperture Radar Interferometry (InSAR) data by Qu et al. (2019)^82^ and Zhang et al. (2022)^83^, as shown in Fig. 11, we performed simulations of Fourier Amplitude Spectrum (FAS), Pseudo-Spectral Acceleration (PSA), and acceleration time series (ACC) for 12 selected stations using the EXSIM12 program^84^ and the inversion parameters for Region B. Three stations (53XQD: GMX-D, *κ*_0_ = 0.0306; 53XXB: GMX-A, *κ*_0_ = 0.0261; 53DFD: GMX-B, *κ*_0_ = 0.0319) were excluded from the GIT framework ​​and used as validation datasets​​, for which GMX-derived average site amplification were applied. For the remaining nine stations (53DFY, 53DZF, 53DHD, 53DSL, 53YRH, 53YBX, 53YLD, 53YPX, 53JYC), the complete simulation parameters, including the source, path, and site models, ​​are systematically presented in Table 3​​.

**Table 3:** Modeling parameters for the stochastic finite-fault simulation of the 2009 *M*_S_ 6.3 yao’an mainshock.

Parameters	Value	Reference
Moment magnitude	Main shock (*M*_w_ = 5.7, SS)	CMT
Rupture location	25.60°N, 101.03°E	This study
Rupture length and width (km)	22 km and 12 km	Qu et al. (2019)
Crustal density (g/cm^3^)	2.8 g/cm^3^	This study
S-wave velocity (km/s)	3.5 km/s	This study
Strike and dip angle	301°, 87°	Qu et al. (2019)
Source slip model	Figure 11	Qu et al. (2019)
Stress drop (MPa)	4.1 MPa	This study
Geometrical spreading	*R*^−1^ for *R* ≤ 75 km 1/75 for 75 < *R* ≤ 130 km (1/75) (130/*R*)^0.5^ for *R* > 130 km	This study
Path duration	0.2*R* for *R* < 50 km 0.075(*R*−50) **+** 10 for 50 ≤ *R* < 90 km −0.003(*R*−90) **+** 13 for 90 ≤ *R* < 250 km 0.064(*R*−250) **+** 12.5 for 250 ≤ *R* < 320 km 0.145(*R*−320) **+** 17 for *R* > 320 km	Fu et al. (2023)
Quality factor	120.26*f* ^0.638^	This study
Site amplification	Supplementary Fig. S2–S5 and Fig. 10	This study

**Fig. 12:**
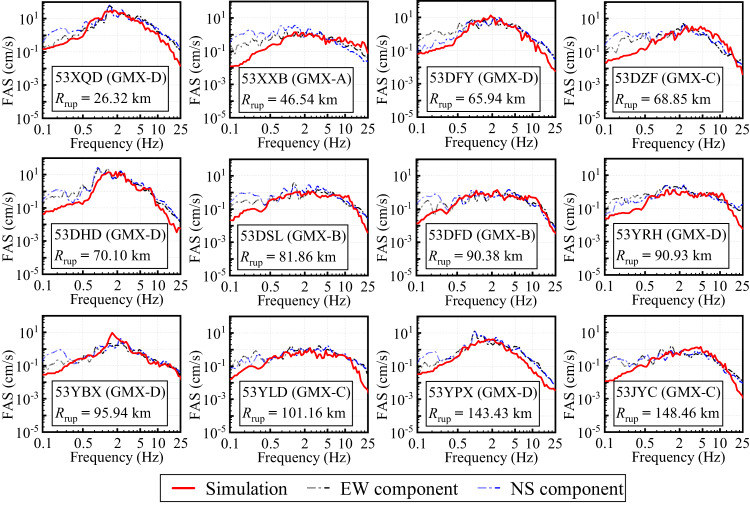
Comparison of the stochastic finite-fault simulated and observed FAS of 12 selected stations.

**Fig. 13:**
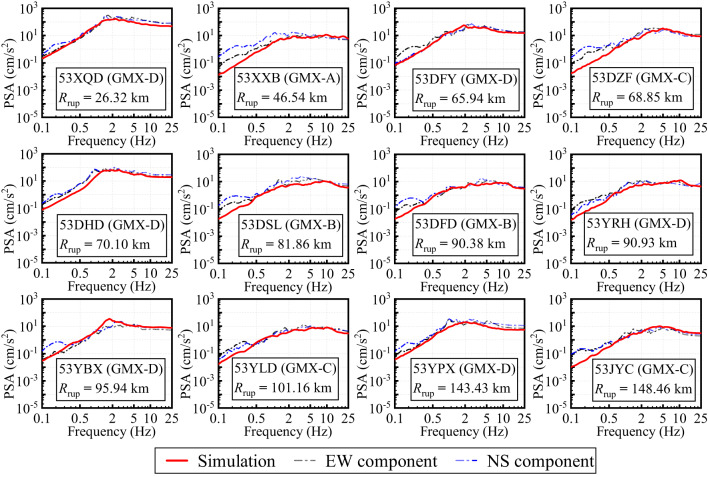
Comparison of the stochastic finite-fault simulated and observed PSA of 12 selected stations.

The 12 selected stations exhibited rupture distances ranging from 26.32 to 148.46 km with a relatively uniform spatial distribution. Figure 12 presents comparative analyses between simulated and observed FAS at these stations. The simulated FAS curves generally reproduce the observed spectral shapes within the frequency band of 0.5–25 Hz. However, systematic underestimations are observed in the low-frequency range (< 0.5 Hz) at nearly all stations. This mismatch may stem from limitations in the stochastic source model regarding long-period energy radiation, as well as from unmodeled long-wavelength path effects and nonlinear site response characteristics under weak versus strong shaking^29^. It is worth noting that even at stations excluded from the inversion process (53XQD, 53XXB, and 53DFD), the simulated FAS curves show excellent agreement with observations above 0.5 Hz, highlighting the robust generalizability of the GMX-based site response model.

The simulated PSA curves also show good consistency with observations within the 0.5–25 Hz frequency range, as shown in Fig. 13. The simulations successfully reproduce the shape and amplitude of spectral peaks, especially at stations such as 53DFY, 53DSL, and 53YLD. This suggests that the finite-fault model, coupled with regionally representative stress drop and attenuation parameters, can effectively capture the energy distribution affecting response spectra across multiple sites. At longer periods (< 0.5 Hz), PSA underestimation becomes more pronounced, consistent with the FAS results. These deviations may also result from the fixed corner frequency and simplified rupture velocity assumptions in the stochastic model, which constrain its ability to generate realistic long-period ground motion. Because the stochastic finite-fault method only incorporates S-waves when generating acceleration time histories, and does not consider the rupture of the fault plane, the propagation of seismic waves in the crust, or the influence of local site conditions on the Fourier phase spectrum, the simulated acceleration time histories cannot reproduce P-waves and surface waves.

## Conclusions

By incorporating station-specific empirical reference site amplification functions into the GIT framework, we inverted source stress drop for 60 events, frequency-dependent quality factors for 4 subdomains and local site effects for 60 strong-motion stations. The logarithmic residuals between observed and modeled FAS exhibit near-Gaussian distributions without systematic biases. Compared to GIT approaches using regionally averaged empirical amplification, our method achieves residual means closer to the theoretical zero-mean condition with significantly reduced standard deviations, suggesting that station-specific empirical reference site amplification functions may enhance inversion constraints and help reduce biases.

Our analysis of 60 small-to-moderate earthquakes (3.0 ≤ *M*_w_ ≤ 6.0) in Yunnan reveals Δ*σ* range from 0.3 to 7.34 MPa, with significant spatial variations. Region C and Region D show the highest average Δ*σ* (2.21 MPa), followed by Region A (2.07 MPa), and Region B (1.16 MPa). High-Δ*σ* events are primarily concentrated near the Sichuan–Yunnan border, aligning with known seismotectonic activity. Based on our limited sample, reverse faulting events exhibit the highest average Δ*σ* (2.74 MPa), followed by strike-slip (1.89 MPa) and normal faulting (1.04 MPa) events, and no significant correlations were found between Δ*σ* and either moment magnitude or focal depth. However, a clear spatial correlation is observed between Δ*σ* and surface heat flow: lower Δ*σ* values are associated with high heat flow regions (Region B: *q*_s_ > 80 mW/m²), while regions with lower heat flow (55–65 mW/m² in Regions A, C, and D) show higher Δ*σ*. This highlights the potential thermomechanical control of crustal temperature on earthquake rupture processes in the region.

The *Q*(*f*) models for the four study areas (A-D) in Yunnan Province were 118.41*f* ^0.691^, 120.26*f* ^0.638^, 77.20*f* ^0.598^ and 72.19*f* ^0.861^, respectively. These results reveal two distinct groups: regions A and B exhibit comparable high *Q*_0_ values, while regions C and D cluster with lower values. This spatial heterogeneity indicates that the Red River Fault Zone serves as a tectonic boundary dividing Yunnan into eastern and western regions, which exhibit significant differences in tectonic activity levels, crustal velocity structure, and geothermal activity states. Overall, the eastern Yunnan region demonstrates higher tectonic activity, lower geothermal heat flow, and faster S-wave attenuation compared to the western region. The site amplifications obtained from HVSR usually do not show a significant high-frequency attenuation effect; this limitation needs to be considered when selecting reference sites based on HVSR technology.

The inverted regional average site amplification models indicate that the peak plateau of the average amplification factor progressively shifts to lower frequencies as the site becomes softer. The *M*_S_ 6.3 Yao’an earthquake was simulated using source, path, and site parameters derived from the GIT inversion. The simulated PSA and FAS exhibited excellent consistency with observational data across the 0.5–25 Hz frequency range, both in spectral shape and amplitude. At frequencies below 0.5 Hz, the slight underestimation of the simulated FAS and PSA may result from limitations in modeling long-period components, fixed corner frequency assumptions, and simplified rupture velocity. Although the simulated acceleration time series did not fully replicate P-wave and surface wave phases, they successfully captured key features of the S-wave components, peak ground acceleration (PGA), and duration. These results validate the reliability of the inversion parameters for Yunnan province.

## Supplementary Information

Below is the link to the electronic supplementary material.

Supplementary Material 1

## Data Availability

The strong-motion datasets used in this study were obtained from the Institute of Engineering Mechanics, China Earthquake Administration, and are not publicly available due to data usage restrictions. However, these data are available from the authors upon reasonable request and with permission of the data provider. For data inquiries or requests, please contact the corresponding author, Prof. Xiaojun Li (beerli@vip.sina.com). All other data generated and analysed during this study, including inversion results and stochastic finite-fault simulation data, are included in this published article and its supplementary information files.
