# Supplementary material for: Ground-motion heterogeneity across four subdomains in Yunnan, China revealed by generalized spectral inversion
Source: Sci Rep. 2025 Nov 18;15:40447. doi: 10.1038/s41598-025-24103-7 (PMC12627742; doi:10.1038/s41598-025-24103-7)
Supplement: Supplementary file 1 — Supplementary Material 1 [file 41598_2025_24103_MOESM1_ESM.docx]

**Table 1.** Number of stations and events in each subdomain (Regions A–D) of Yunnan Province.

| Region | No. of stations | No. of events |
| --- | --- | --- |
| A | 14 | 14 |
| B | 24 | 25 |
| C | 10 | 6 |
| D | 17 | 15 |

**Table 2.** Site definition of the Geomatrix Site Classification (GMX).

| Site class | Description |
| --- | --- |
| A | Rock: instruments are placed on rock with S-wave velocity greater than 600 m/s or on sites with less than 5 m of soil over rock. |
| B | Shallow (stiff) soil: instruments are placed on sites with a soil profile no more than 20 m thick overlying rock |
| C | Deep narrow soil: instruments are placed in narrow valleys or canyons less than a few kilometers wide with a soil profile over 20 m thick overlying rock. |
| D | Deep broad soil: instruments are placed in broad valleys with a soil profile over 20 m thick overlying rock. |
| E | Deep soft soil: instruments are placed on sites with a deep soil profile with an average S-wave velocity less than 150 m/s. |

Equation (3.6) can be reformulated in matrix notation as follows, where *q_k_* = *-πfR_ij_*/(ln(10)*β*). The *w* is used as a linearity constraint on the quality factor, by requiring the second-order numerical derivative to be zero.

$$\left(\begin{matrix} 1 & & & & & & & & & & & & q_{1} & & & 1 & & & & & & \\ & \ddots& & & & & & & & & & & & \ddots& & & \ddots& & & & & \\ & & 1 & & & & & & & & & & & & q_{k} & & & 1 & & & & \\ \vdots& \vdots& \vdots& & & & & & & & & & \vdots& \vdots& \vdots& & & & \ddots& & & \\ 1 & & & & & & & & & & & & q_{1} & & & & & & & 1 & & \\ & \ddots& & & & & & & & & & & & \ddots& & & & & & & \ddots& \\ & & 1 & & & & & & & & & & & & q_{k} & & & & & & & 1 \\ & & & 1 & & & & & & & & & q_{1} & & & 1 & & & & & & \\ & & & & \ddots& & & & & & & & & \ddots& & & \ddots& & & & & \\ & & & & & 1 & & & & & & & & & q_{k} & & & 1 & & & & \\ & & & \vdots& \vdots& \vdots& & & & & & & \vdots& \vdots& \vdots& & & & \ddots& & & \\ & & & 1 & & & & & & & & & q_{1} & & & & & & & 1 & & \\ & & & & \ddots& & & & & & & & & \ddots& & & & & & & \ddots& \\ & & & & & 1 & & & & & & & & & q_{k} & & & & & & & 1 \\ & & & & & & \ddots& \ddots& \ddots& & & & \vdots& \vdots& \vdots& \vdots& \vdots& \vdots& \vdots& \vdots& \vdots& \vdots\\ & & & & & & & & & 1 & & & q_{1} & & & 1 & & & & & & \\ & & & & & & & & & & \ddots& & & \ddots& & & \ddots& & & & & \\ & & & & & & & & & & & 1 & & & q_{k} & & & 1 & & & & \\ & & & & & & & & & \vdots& \vdots& \vdots& \vdots& \vdots& \vdots& & & & \ddots& & & \\ & & & & & & & & & 1 & & & q_{1} & & & & & & & 1 & & \\ & & & & & & & & & & \ddots& & & \ddots& & & & & & & \ddots& \\ & & & & & & & & & & & 1 & & & q_{k} & & & & & & & 1 \\ 0 & \cdots& 0 & 0 & \cdots& 0 & \cdots& \cdots& \cdots& 0 & \cdots& 0 & 0 & \cdots& 0 & 1 & \cdots& 1 & \cdots& 1 & \cdots& 1 \\ 0 & \cdots& 0 & 0 & \cdots& 0 & \cdots& \cdots& \cdots& 0 & \cdots& 0 & -2w & \cdots& 0 & 0 & \cdots& 0 & \cdots& 0 & \cdots& 0 \\ 0 & \cdots& 0 & 0 & \cdots& 0 & \cdots& \cdots& \cdots& 0 & \cdots& 0 & 0 & \ddots& 0 & 0 & \cdots& 0 & \cdots& 0 & \cdots& 0 \\ 0 & \cdots& 0 & 0 & \cdots& 0 & \cdots& \cdots& \cdots& 0 & \cdots& 0 & 0 & \cdots& -2w & 0 & \cdots& 0 & \cdots& 0 & \cdots& 0 \end{matrix}\right)\left(\begin{matrix} \log\left(E_{1}\left(f_{1}\right)\right) \\ \log\left(E_{1}\left(f_{2}\right)\right) \\ \vdots\\ \log\left(E_{1}\left(f_{k}\right)\right) \\ \log\left(E_{2}\left(f_{1}\right)\right) \\ \log\left(E_{2}\left(f_{2}\right)\right) \\ \vdots\\ \log\left(E_{2}\left(f_{k}\right)\right) \\ \vdots\\ \log\left(E_{i}\left(f_{k}\right)\right) \\ 1/Q\left(f_{1}\right) \\ 1/Q\left(f_{2}\right) \\ \vdots\\ 1/Q\left(f_{k}\right) \\ \log\left(S_{1}\left(f_{1}\right)\right) \\ \log\left(S_{1}\left(f_{2}\right)\right) \\ \vdots\\ \log\left(S_{1}\left(f_{k}\right)\right) \\ \log\left(S_{2}\left(f_{1}\right)\right) \\ \log\left(S_{2}\left(f_{2}\right)\right) \\ \vdots\\ \log\left(S_{2}\left(f_{k}\right)\right) \\ \vdots\\ \log\left(S_{j}\left(f_{k}\right)\right) \end{matrix}\right)=\left(\begin{matrix} FAS_{11}\left(f_{1}\right) \\ FAS_{11}\left(f_{2}\right) \\ \vdots\\ FAS_{11}\left(f_{k}\right) \\ FAS_{12}\left(f_{1}\right) \\ FAS_{12}\left(f_{2}\right) \\ \vdots\\ FAS_{12}\left(f_{k}\right) \\ \vdots\\ \vdots\\ FAS_{ij}\left(f_{k}\right) \\ 0 \\ \vdots\\ 0 \end{matrix}\right)$$

**Fig. 1.** The Community Velocity Model V.2.0 (CVM-2.0) of the SYB (Liu et al., 2023).

**Fig. 2.** Site amplifications obtained from the GIT, HVSR and A( *f* )exp(-π*fκ*_0_) for 14 stations in Region A.

**Fig. 3.** Site amplifications obtained from the GIT, HVSR and A( *f* )exp(-π*fκ*_0_) for 24 stations in Region B.

**Fig. 4.** Site amplifications obtained from the GIT, HVSR and A( *f* )exp(-π*fκ*_0_) for 5 stations in Region C.

**Fig. 5.** Site amplifications obtained from the GIT, HVSR and A( *f* )exp(-π*fκ*_0_) for 17 stations in Region D.

Supplementary Figures S2–S5 present the inverted site amplification curves of the selected 60 strong-motion stations for 4 subdomains. These curves are compared with those derived from the HVSR method and *A*( *f* )exp(-π*fκ*_0_). In region C, five stations (53BWL、53CBG、53CMX、53ELK、53HTJ) were excluded due to insufficient ground-motion data, which led to non-convergent site amplification solutions and physically implausible amplification factors at the predominant frequencies. Except for stations 53DSL, 53JYC, 53XXZ, 53YPX, and 53DJL, the inverted site amplification curves obtained from the GIT and HVSR method exhibit similar shapes, accurately capturing the predominant frequency of each station. In contrast, *A*( *f* )exp(-π*fκ*_0_) represents the empirical reference site amplification derived from *V*_s30_ velocity profiles, which does not incorporate local topographical effect, basin effect, or energy loss due to reflection, refraction, and scattering.

**Fig. 6.** Comparison of the stochastic finite-fault simulated and observed horizontal acceleration time series of 12 selected stations.

Supplementary Figures S6 shows the observed and simulated acceleration time histories in two horizontal directions at 12 stations. The results demonstrate that the simulated acceleration time histories closely resemble the observed records in terms of S-wave content, peak ground acceleration (PGA), and duration.
